# Challenges in understanding common disease

**DOI:** 10.1186/s13073-017-0506-1

**Published:** 2017-12-18

**Authors:** Peter M. Visscher

**Affiliations:** Institute for Molecular Bioscience and Queensland Brain Institute, University of Queensland, St Lucia, QLD 4072 Australia

## Abstract

Peter M. Visscher discusses advances in our understanding of complex disease, the challenges in applying this knowledge to functional follow-up, and the potential implications for therapeutic interventions.

## Introduction

**Fig. 1:**
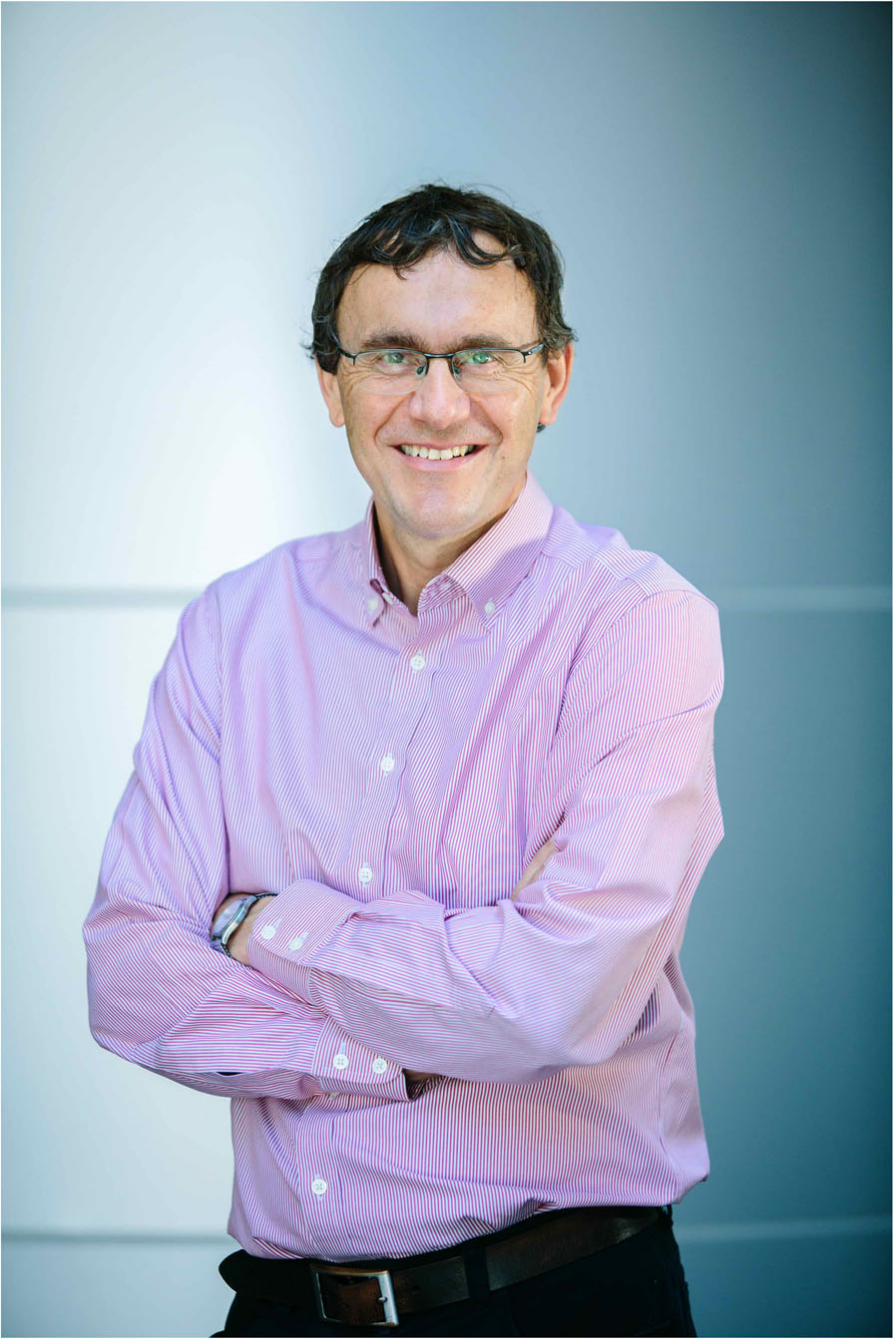
Peter M. Visscher

Peter M. Visscher (Fig. 1) is Professor of Quantitative Genetics and Director of the Program in Complex Trait Genomics at the University of Queensland. He is a Senior Principal Research Fellow of the Australian National Health and Medical Research Council and an elected Fellow of the Australian Academy of Science. His research interests are focused on genetic variation for complex traits in common disease, and on systems genomics. In this Q&A, he shares his insights on understanding complex disease and the future of this exciting field of research.

## How did your interest in complex diseases begin?

My early career was in agricultural (animal) genetics, where ‘complex traits’ are quantitative traits of economic importance for breeders and farmers, for example milk production in dairy cows and growth rates in pigs. Training in animal genetics (breeding) provides an excellent springboard for human complex disease genetics because quantitative genetics is steeped in a century worth of strong theory combined with sophisticated statistical genetic analysis methods for the estimation and prediction of genetic values in large datasets. In 1996, when I was working at the University of Edinburgh, I was alerted to a paper from local colleagues in psychiatric genetics who were working on bipolar disorder and schizophrenia in large human pedigrees that contained over 100 individuals [1]. I thought that my background and expertise could lead to a fruitful collaboration, and it did. The first paper from that collaboration [2] set the scene for my subsequent research on a more polygenic approach to disease mapping for complex traits, by modelling disease as a quantitative trait. This positive experience and the realisation that I might be able to contribute something useful and make discoveries in a different field of research led me to switch from animal to human genetics. I have never regretted that move.

## Why is this area of research so important at the present time?

Common diseases, such as cardiovascular disease, type 2 diabetes, psychiatric disorders, cancers, and auto-immune disorders, create the largest burden on society, both at a personal level and economically. We know from many different kinds of genetic studies that a substantial proportion of individual differences in susceptibility to disease are genetic, so genetics is one way of understanding the biology and ultimately to approach the prevention and better treatment of genetic disorders. We have the genomic technology available to tackle common disease and so the time to potentially make real progress is now.

## What are the main challenges in understanding complex diseases such as psychiatric and neurological disorders?

Some of the most pressing challenges are limitations to the experimental sample size and the depth of information on environmental exposures and disease-associated phenotypes. Common diseases are characterised by the interplay of genetic and environmental factors and only by measuring both can we fully dissect disease and separate cause from consequence. We now have the tools to affordably interrogate the entire genome with a single nucleotide polymorphism (SNP) chip or more expensively using whole-genome sequencing (WGS), but measuring lifetime environmental factors or exposures with similar depth is in its infancy. Examples of such factors and exposures are stressful life events, sleep patterns, diet, the microbiome, and social networks. In all of these studies, sample size is key, because it is unequivocal that, on average, the contribution of individual genetic risk loci and individual environment exposures on disease risk are small.

Another challenge is widespread pleiotropy (genetic effects on multiple traits and diseases). Research in the past 5 years has revealed genetic overlap between psychiatric disorders (e.g. schizophrenia and major depression), between neurological disorders, and between psychiatric and neurological disorders. This genetic overlap tells us that nature is not obeying traditional diagnostic boundaries of diseases and disorders. By measuring multiple disease-associated phenotypes and objective symptoms in a consistent manner and on a large scale, we would have unbiased empirical data to better understand the path from genome to phenome. This may lead to a radical change in diagnosis and, perhaps, treatment.

If we were to have the same depth of information on a large number of individuals with diseases as are measured in national biobanks such as the UK Biobank [3], the Estonia Biobank [4], and Lifelines (Netherlands) [5] then mental health research would take a huge leap forward. I am not aware of a planned study that is sufficiently large (millions of individuals) to create a rich databank that is relevant for multiple diseases with a prevalence of about 1%. Ideally, such a study would be at the scale of an entire country (for example the UK Biobank scaled up). Although such a study might seem expensive, I am convinced it would be a worthwhile economic investment for tax payers.

## Which tools are most important to understand the mechanisms that underlie complex diseases?

For the discovery of genes, gene variants, and biological pathways, SNP arrays still far outweigh the benefits of WGS. It is a simple sum: SNP arrays followed by imputation to a large fully-sequenced reference panel provide most of the information on genotype–phenotype association at about 5% of the cost of WGS, at today’s prices. As sample size is still limiting discovery, it would make much more sense to SNP-chip 100,000 individuals than to sequence a few thousand. If in the future the price differential is small, then it would make sense to obtain full sequences.

Overlaying gene-trait association data with other data resources such as gene expression and epigenetic annotation has already proved successful to understand complex disease mechanisms in a growing number of examples (e.g. [6]). In my opinion, a current challenge is to experimentally perturb the genome at many loci simultaneously (to reflect the real polygenic nature of disease) in a high-throughput manner. The rapid development and uptake of gene editing technologies holds promise to do this, perhaps in tandem with organs-on-a-chip tools. My prediction is that, increasingly, human cells and tissues will be the basis of experimental studies, rather than traditional experimental organisms.

## What are the challenges of taking polygenic disease forward to functional follow-up?

There are rapid developments in analytical tools that exploit the availability of data from large genome-wide association studies (GWAS). Data from community resources such as GTEx, the CommonMind Consortium, and epigenetic datasets can be used to identify the likely target genes that explain SNP–disease associations. These software tools generate hypotheses about function (e.g. SNP - > methylation - > gene expression - > disease risk) and also about possible drug targets that could, in principle, be tested experimentally. However, what we do not know is which experimental framework to use to test the many hypotheses that are generated by the analysis tools.

## What do you consider to be the most promising approaches for clinical treatment of complex diseases and how do we target these therapeutically?

Genetic studies such as GWAS generate knowledge in a number of different ways that might lead to prevention or treatment. Because of the huge sample sizes of GWAS, genetic risk predictors based on SNP chip data are becoming more accurate and are starting to identify individuals who are at high risk of, for example, type-2 diabetes, inflammatory bowel disease, or schizophrenia. With ever increasing sample sizes, the genetic information could be used in the future as predictors to help with patient diagnosis, e.g. between Crohn’s disease and ulcerative colitis, or between schizophrenia and bipolar disorder. These predictors could lead to improved diagnosis and early therapeutic intervention.

Genetic studies are also increasingly generating hypotheses about possible drug targets or drug repurposing, and major pharmaceutical companies are starting to use the results from GWAS and other genetic findings in their decision making [7]. I expect this process to accelerate in the near future, in that researchers will discover more frequent occurrences of small genetic perturbations that lead to increased risk of disease through an established mechanism (e.g. mediated by gene expression), and that there may be existing or promising chemical perturbations that can alter the risk of disease.

## What are your thoughts on the recently proposed omnigenic model of complex disease and its implications for clinical therapies?

I do not quite understand the fuss created by the publication of the scholarly perspectives article by Boyle and colleagues [8]. Perhaps it is my background in quantitative genetics, but we have known for many years that complex traits and common diseases are highly polygenic and pleiotropic. The fundamental questions for the development of clinical therapies are how and where to perturb the genome to bring about a beneficial outcome.

Take the example of statins. On one hand, statins are a very successful group of lipid-lowering medications and they reduce the risk of cardiovascular disease (CVD). On the other hand, genetic research on either lipids or CVD has shown that variation in these traits in the population is both polygenic and pleiotropic [9]. Clearly, these facts are not mutually exclusive; widespread polygenicity and pleiotropy do not preclude effective therapy.

## How do you see the area of polygenic disease research developing in the next 5 to 10 years?

Predictions of developments are difficult, in particular a prediction about 10 years from now. Few, if any, would have predicted only 10 years ago the enormous range of discoveries made through the experimental design of GWAS.

Disruptive initiatives such as the UK Biobank [3] and, hopefully, the All-of-Us initiative in the USA [10], together with emerging initiatives in China, will accelerate discoveries of genes, gene variants, and biological pathways in polygenic disease. These endeavours may help to tease apart the role of genetic and environmental factors and firmly anchor observational data on associations between traits in cause and effect. New discoveries are likely to be aided by new high-throughput technologies to measure environmental exposures in real time via smart devices (e.g. [10]).

Sequencing technologies will likely continue to develop and reduce in cost so that perhaps, in 5 to 10 years from now, whole-genome surveys of genetic variation on sample sizes in the order of millions will be done by WGS instead of SNP chips. I predict that stacking data from different sources will lead to the identification of an increasing number of causal variants for common diseases and an increasing number of genes for which the evidence is strong that they are the target of discovered SNP–trait associations. Genetic predictors for some traits and diseases will become sufficiently powerful to be used in clinical settings. Genetic discoveries will lead to new and successful drug trials, when chemical perturbations mimic the action of environmental or genetic perturbations. Finally, I hope that high-throughput multiple locus experimental perturbation techniques will mature so that polygenic variation can be studied at the cellular level.

## Abbreviations

CVD: Cardiovascular disease
GWAS: Genome-wide association studies
SNP: Single nucleotide polymorphism
WGS: Whole-genome sequencing
